# Facile and Green Synthesis of Graphene-Based Conductive Adhesives via Liquid Exfoliation Process

**DOI:** 10.3390/nano9010038

**Published:** 2018-12-28

**Authors:** Jhao-Yi Wu, Yi-Chin Lai, Chien-Liang Chang, Wu-Ching Hung, Hsiao-Min Wu, Ying-Chih Liao, Chia-Hung Huang, Wei-Ren Liu

**Affiliations:** 1 Department of Chemical Engineering, Chung Yuan Christian University, R&D Center for Membrane Technology, 32023, No. 200, Chun Pei Rd., Chung Li District, Taoyuan 32023, Taiwan; james19931201@gmail.com; 2 Department of Chemical Engineering, National Taiwan University, No. 1, Sec. 4, Roosevelt Rd., Taipei 10617, Taiwan; yijin10221339@gmail.com; 3 National Chung Shan Institute of Science & Technology, Neighborhood, Sec. Jia’an, Zhongzheng Rd., Longtan Dist., Taoyuan 32546, Taiwan; rodin2005@yahoo.com.tw (C.-L.C.); 5000blackblue@gmail.com (W.-C.H.); prettysandy119@gmail.com (H.-M.W.); 4 Metal Industries Research and Development Centre, Kaohsiung 81160, Taiwan; chiahung@mail.mirdc.org.tw

**Keywords:** graphene, liquid exfoliation, polyvinylidene fluoride, conductive adhesives, flexiable

## Abstract

In this study, we report a facile and green process to synthesize high-quality and few-layer graphene (FLG) derived from graphite via a liquid exfoliation process. The corresponding characterizations of FLG, such as scanning electron microscopy (SEM), transmission electron microscope (TEM), atomic force microscopy (AFM) and Raman spectroscopy, were carried out. The results of SEM show that the lateral size of as-synthesized FLG is 1–5 μm. The results of TEM and AFM indicate more than 80% of graphene layers is <10 layers. The most surprising thing is that D/G ratio of graphite and FLG are 0.15 and 0.19, respectively. The result of the similar D/G ratio demonstrates that little structural defects were created via the liquid exfoliation process. Electronic conductivity tests and resistance of composite film, in terms of different contents of graphite/polyvinylidene difluoride (PVDF) and FLG/PVDF, were carried out. Dramatically, the FLG/PVDF composite demonstrates superior performance compared to the graphite/PVDF composite at the same ratio. In addition, the post-sintering process plays an important role in improving electronic conductivity by 85%. The composition-optimized FLG/PVDF thin film exhibits 81.9 S·cm^−1^. These results indicate that the developed FLG/PVDF composite adhesives could be a potential candidate for conductive adhesive applications.

## 1. Introduction

Electronics or microelectronics industries have transitioned into making electronics lighter, smaller, thinner and more highly efficient [1,2]. Aside from this, they must employ techniques and materials that are environmentally friendly. As a result, they opt to replace lead-based solders for microelectronics packaging with electrically conductive adhesives (ECAs). A great deal of research has been carried out on the fabrication of polymer composites reinforced by metallic fillers ranging from metallic particles to carbon materials. These polymer composites have been used to develop new types of ECAs to reduce the cost to provide finer pitch capability [3]. Gold, silver [4,5,6,7], copper [8], palladium and nickel have been widely used as fillers for commercial ECAs due to their stability and excellent electrical conductivity [7,9]. However, a high concentration of filler is required to form a good electrical conductivity [5], which is the main reason for the high cost of ECAs. How to reduce the loading of metal fillers into the ECAs has become an important issue.

Many researches use carbon nanotubes (CNTs) [10,11,12] as fillers to improve the electrical properties of ECAs due to their high conductivity, flexibility, and large aspect ratios, and report the establishment of a percolated network at low filler content. However, the usage of CNTs has been limited by the challenges in processing, high cost and dispersion [13]. As a two-dimensional material, graphene has attracted wide attention due to its stable structure and excellent performance. These materials have drawn an intensive attention towards a variety of research fields to utilize their exceptional thermal, mechanical, optical and electrical properties [14,15,16]. Theoretical and experimental studies of graphene show they may possess high thermal conductivity (5000 W·m^−1^·K^−1^), high Young’s modulus (~1 TPa), large surface area (~2600 m^2^·g^−1^) [17,18] and great electrical conductivity (6000 S·cm^−1^) [19]. Possessing the extremely high aspect ratio among all the nanostructured materials, graphene is a promising nanomaterial able to establish a percolated network at very low concentrations.

Xie et al. predicted that in comparison with nanotubes, graphene could provide higher conductivity when used as fillers for conductive adhesives. This is due to its large specific area [20,21,22]. Electrical conductivities of auxiliary forms of graphene have also been reported and studied as conductive fillers or reinforcements for polymers. The polypropylene/graphene oxide (PP/GO) nanocomposite prepared by Huang and colleagues through Zieglar-Natta polymerization provides a high conductivity of 0.3 S·m^−1^ at a 4.9 wt.% loading of GO [23,24]. The chemically reduced graphene oxide/polystyrene (CRGO/PS) composite prepared by Stankovich [25] delivers 0.1 S·m^−1^. Qi et al. [26] compared the electrical conductivity of MWCNT/PS and G/PS nanocomposites and found that at 0.69 wt.%, G/PS has a conductivity of 3.49 S·m^−1^ while MWCNT/PS at the same content only has a conductivity of 3 × 10^−5^ S·m^−1^. Ansari et al. studied and compared the properties of functionalized graphene sheet/PVDF (FGS/PVDF) and exfoliated graphite/PVDF (EG/PVDF), which were prepared via a solution process with subsequent compression to fabricate conductive nanocomposites. According to their results, FGS remained well dispersed in PVDF and showed wrinkled topography with relatively thin graphene sheets that were bonded well in the matrix [27].

In order to obtain graphene, various methods, such as exfoliation and cleavage, liquid-phase exfoliation [28,29], growth on SiC [30,31], chemical vapor deposition [32,33], molecular beam epitaxy, chemical synthesis [34,35,36,37], and chemical routes, have been widely studied. Mass production of single-layer graphene or few-layer graphene is being hindered by the expensive cost and environmental threat of its conventional synthesis. From the environmentally friendly viewpoint, there are many researches that have studied the synthesis of graphene by a simple ultrasonication or jet cavitation method to obtain few-layer graphene [38]. In this paper, a low-temperature, high-pressure continuous flow cell disrupter was used as a delamination device to synthesize few-layer graphene. With this method, simple, scalable, low-cost graphene was swiftly produced without the incorporation of any toxic chemicals. The graphene obtained by this method was combined with polymer to make ECAs.

## 2. Experimental

### 2.1. Synthesis of FLG

Natural flake graphite (Øave = 500 µm) was used as a feed material for the delamination process while *N*-Methyl-2-pyrrolidone (NMP) and polyvinylidene fluoride (PVDF) purchased from UBIQ Technology Co. Ltd., Taiwan were used as a dispersing agent and a binder, respectively. The synthesis of FLG was described as follow: a 1 kg solution consisting of 10 wt.% natural graphite dispersed in de-ionized water (DI H_2_O) was fed into a low-temperature, ultra-high-pressure continuous flow cell disrupter (LTHPD, JNBIO, JN 10C, Guangzhou, China). Prior to feeding the solution into the tank of the LTHPD, the solution was vigorously stirred to ensure uniform dispersion. The graphite solution was pumped into a nozzle with a high pressure and a high flow rate. Under the high-pressure, strong-impact and repeated cyclic stress, layers of graphite were exfoliated, obtaining few-layer graphene (FLG). LTHPD did not only delaminate the graphite flakes into FLG, but also ensures homogeneous dispersion in the solution. The operating condition was maintained at 1800 bar and 14–16 °C. The obtained product was dried in a vacuum oven at 30 °C overnight.

### 2.2. Conductive Adhesive Slurry Preparations

Different slurry compositions were prepared with various FLG:PVDF ratios, such as 1:99, 5:95, 8:92, 10:90, 30:70, 90:90, and 50:50 by dispersing PVDF with a designated amount into NMP. After ensuring the homogeneity of PVDF in NMP, FLG was added into the solution and stirred for 2 h. The slurry was coated onto glass slides and dried at 80 °C for 1 h. The graphite:PVDF slurry of the same compositions was also prepared for comparison.

### 2.3. Characterizations

The height profile of as-synthesized few-layer graphene was measured by using atomic force microscopy (AFM, Bruker Dimension Icon). The samples for AFM were prepared by dropping the dispersion directly onto a freshly cleaved mica wafer. Raman spectra were carried out by a micro Raman spectroscopy system with a laser frequency of 532 nm as the excitation source. The morphologies of the samples were analyzed using scanning electron microscopy (SEM) by Hitachi S-4100 (Hitachi, Tokyo, Japan) and high-resolution transmission electron microscopy (HRTEM) JEOL-JEM2000FXII (JEOL, Tokyo, Japan). The electrical conductivities of the graphene adhesives were measured using a resistivity meter (KeithLink TG2, KeithLink, Taipei, Taiwan) with a four-point probe.

## 3. Results and Discussion

### 3.1. Characterizations of FLG

**Figure 1:**
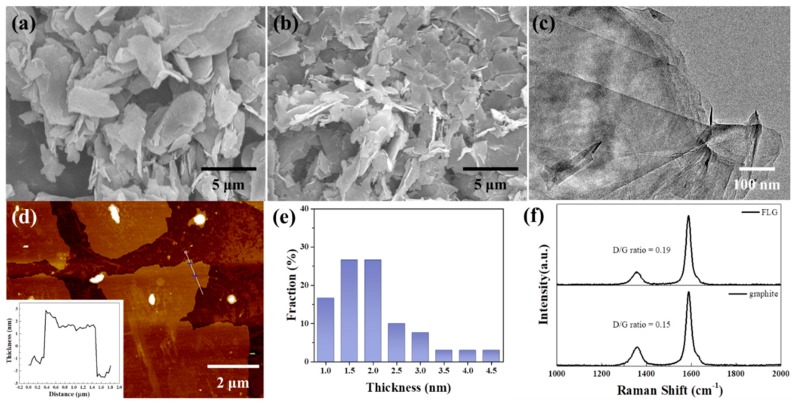
SEM images of (**a**) graphene and (**b**) FLG; (**c**) TEM image of FLG; (**d**) AFM image of FLG, insert: height profiles of FLG; (**e**) thickness distribution of FLG measured by AFM on 35 samples; (**f**) Raman spectra of graphite and FLG.

Figure 1a,b show images of graphite and FLG with lateral sizes of 5–10 μm and 1–5 μm, respectively. After treatment by LTHPD, the graphite size was decreased and delaminated to be FLG. This shows that the thick sheets of graphite can be effectively exfoliated into thinner sheets. Figure 1c displays the HRTEM image of the FLG. This image of FLG is transparent and folded, which coincides well with the typical feature of the reported FLG. In order to observe the effect of the LTHPD, AFM characterization was carried out for its convenience in measuring flake thickness. The FLG was deposited on the silica wafer and dried in room temperature. More than 30 flakes were measured to determine the distribution. Figure 1d,e show the AFM image and the thickness distribution of FLG. They are believed to be monolayers according to the fact that FLG are often measured to be 0.4–1 nm by AFM due to some external factors such as the AFM equipment and substrates [39,40]. This shows that the range of FLG thickness is 1–4.5 nm, which is less than 10 layers. The delamination process used to obtain FLG was expected to cause defects, and thus, Raman analysis of graphite and FLG was determined and is depicted in Figure 1f. The result of similar D/G ratio demonstrates that little structural defects were created by the liquid exfoliation process. Additionally, the intensity ratio of D/G for the FLG is 0.19, which is much lower than that of the GO and chemically reduced graphene [41,42].

### 3.2. Characterizations of Conductive Adhesives

**Figure 2:**
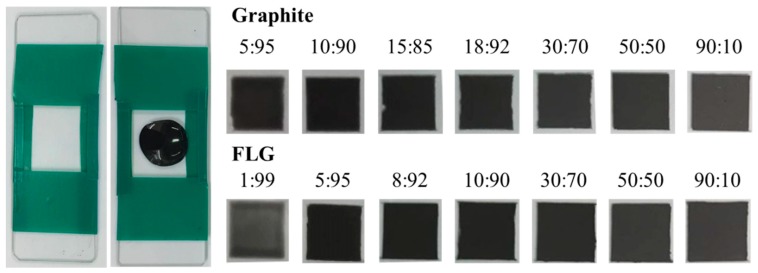
Photo images of graphite/PVDF and FLG/PVDF composites.

**Figure 3:**
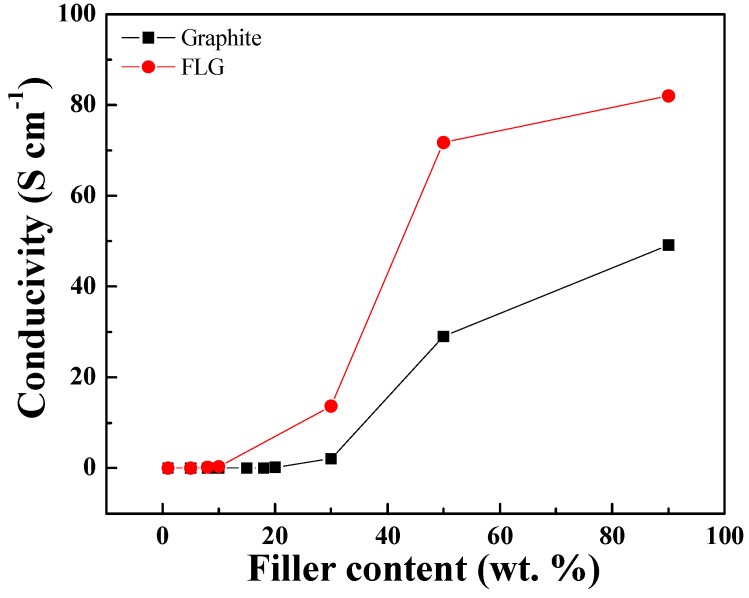
Electronic conductivity of graphite/PVDF and FLG/PVDF composite films measured by a four-point probe.

To formulate conductive adhesives, the conductive fillers, graphite and FLG were mixed with PVDF. After mixing, the composites inks were cast into a 2 × 2 mm well to create solid thin films (Figure 2). The dried thin films at various filler/PVDF ratios exhibit color variations. The FLG/PVDF composite also exhibits great conductivity compared to its graphite counterpart. As shown in Figure 3, at the same filler/PVDF ratio, the conductivity of the FLG/PVDF composite is always higher than that of the graphite/PVDF composite, which is possibly due to the higher aspect ratio of FLG. The lateral size of FLG is ~8 μm and the average thickness of FLG is ~4 nm. Thus, the aspect ratio of as-synthesized FLG is as high as ~2000. The higher aspect ratio provided a better percolation network for electron transfer, and thus resulted in the conductivity enhancement.

**Figure 4:**
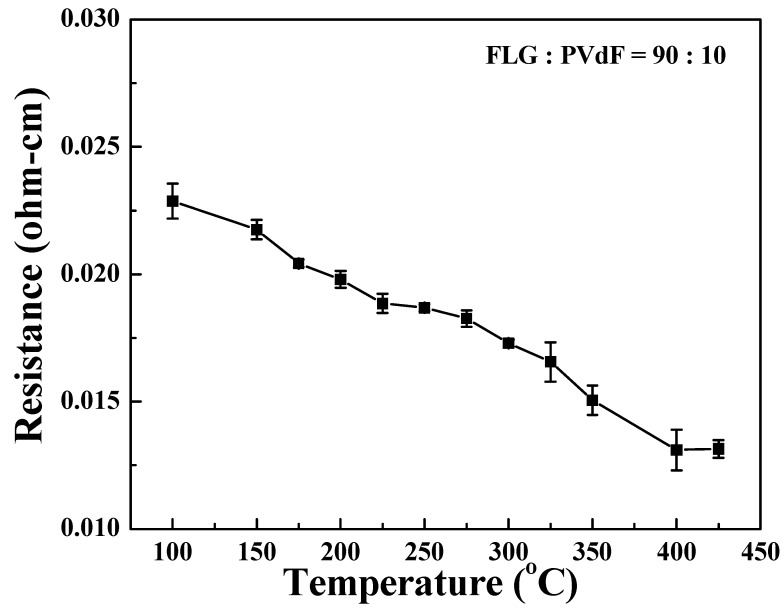
Variation in sheet resistance of FLG/PVDF composite films with temperature. The FLG/PVDF thin film is coated on glass with a thickness of 0.02 mm and is placed on a hot plate for 30 min.

The conductivity of the FLG/PVDF thin film can be greatly enhanced after sintering. As shown in Figure 4, when the temperature increases, the resistance decreases. This reduction of resistivity comes from relaxation of the polymer chains at higher temperatures and thus leads to a decrease in the mean distance between graphene [43,44,45]. The shortened distance between graphene layers helps the electron transfer and thus increases the conductivity [46]. However, as the temperature reaches the glass transition temperature of PVDF from ~400 to 425 °C, the relaxation process facilitates the re-arrangement of the dispersed graphene [45] and thus the resistance plateau occurs at ~400 °C.

**Figure 5:**
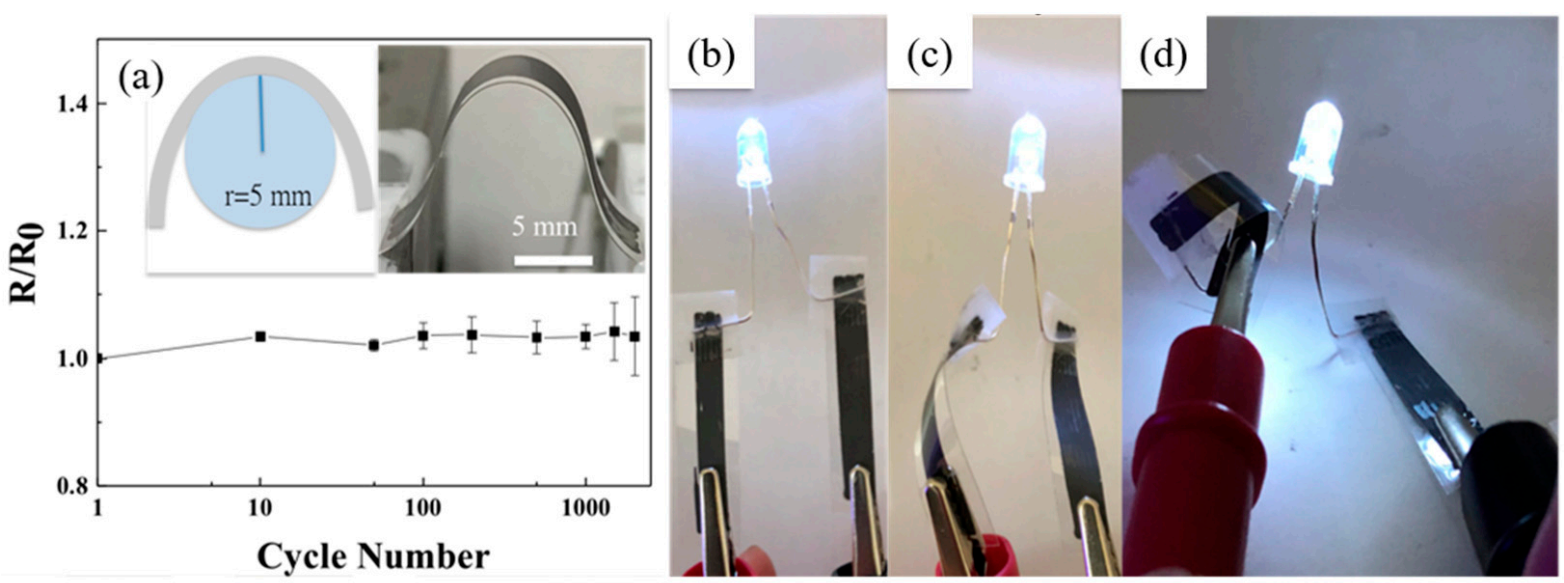
(**a**) The resistance increase ratio (R/R_0_) of graphene/PVDF thin film on the PET film under the bending performance test with a radius of curvature of 5 mm. After connecting the graphene/polymer composite to an LED, the light remained bright at various degrees of bending: (**b**) flat, (**c**) bending, and (**d**) spiral shape.

The FLG/PVDF composite also exhibits great flexibility and mechanical strength after being coated on flexible plastic sheets. The electrical resistance of the FLG/PVDF thin film on the PET film under the bending test (inset picture in Figure 5a) shows a fairly small variation after thousands of bending cycles. The small increase of ~5% in resistance is possibly attributed to the crack of film under bending [47]. This great mechanical strength makes the FLG/PVDF a great conductive adhesive for flexible electronic applications. For demonstration, two pieces of printed FLG/PVDF thin films on the PET film were connected to an LED, and the printed conductive tracks were twisted or bent with different degrees of curvature (Figure 5b,d). As shown in Figure 5c, the PET film with slight curvature could remain bright. The bulb shone constantly after bending the film into a spiral shape, as shown in Figure 5d, indicating the great bending property of the graphene/PVDF composites on the PET film.

## 4. Discussion

In this study, a facile liquid exfoliation process was developed to synthesize FLG. Electron microscopic examination shows the as-synthesized FLG has a lateral size of 1–5 μm. AFM indicates more than 80% of FLG has less than 10 layers. Raman spectroscopy also shows a great D/G ratio of 0.19, which is close to that of graphite (0.15), indicating that little structural defects were created via the liquid exfoliation process. After being mixed with PVDF, the composite FLG/PVDF thin film exhibits superior electronic conductivity compared to the graphite/PVDF composite with the same ratio. Moreover, the post-sintering process can further improve electronic conductivity by 85%. The composition-optimized FLG/PVDF composite exhibits 81.9 S·cm^−1^. The printed FLG/PVDF thin film patterns also show great flexibility and mechanical strength under bending conditions. The electrical conductivity remains nearly the same after thousands of bending cycles. These results indicate that the formulated FLG/PVDF composite adhesives have a great potential in conductive adhesive applications.
